# Neuronal Activation in the Central Nervous System of Rats in the Initial Stage of Chronic Kidney Disease-Modulatory Effects of Losartan and Moxonidine

**DOI:** 10.1371/journal.pone.0066543

**Published:** 2013-06-20

**Authors:** Miklós Palkovits, Katarína Šebeková, Kristina Simon Klenovics, Anton Kebis, Gholamreza Fazeli, Udo Bahner, August Heidland

**Affiliations:** 1 Neuromorphological and Neuroendocrine Research Laboratory, Semmelweis University and the Hungarian Academy of Sciences, Budapest, Hungary; 2 Institute of Molecular Biomedicine, Medical Faculty, Comenius University, Bratislava, Slovakia; 3 Institute of Physiology, Medical Faculty, Comenius University, Bratislava, Slovakia; 4 Laboratory of Organ Perfusion of Slovak Center of Organ Transplantation, Slovak Medical University, Bratislava, Slovakia; 5 Institute of Pharmacology and Toxicology, University of Wuerzburg, Wuerzburg, Germany; 6 KfH-Kidney Centre, Wuerzburg, Germany; 7 Department of Internal Medicine, University of Wuerzburg and KfH-Kidney Centre, Wuerzburg, Germany; University of Houston, United States of America

## Abstract

The effect of mild chronic renal failure (CRF) induced by 4/6-nephrectomy (4/6NX) on central neuronal activations was investigated by c-Fos immunohistochemistry staining and compared to sham-operated rats. In the 4/6 NX rats also the effect of the angiotensin receptor blocker, losartan, and the central sympatholyticum moxonidine was studied for two months. In serial brain sections Fos-immunoreactive neurons were localized and classified semiquantitatively. In 37 brain areas/nuclei several neurons with different functional properties were strongly affected in 4/6NX. It elicited a moderate to high Fos-activity in areas responsible for the monoaminergic innervation of the cerebral cortex, the limbic system, the thalamus and hypothalamus (e.g. noradrenergic neurons of the locus coeruleus, serotonergic neurons in dorsal raphe, histaminergic neurons in the tuberomamillary nucleus). Other monoaminergic cell groups (A5 noradrenaline, C1 adrenaline, medullary raphe serotonin neurons) and neurons in the hypothalamic paraventricular nucleus (innervating the sympathetic preganglionic neurons and affecting the peripheral sympathetic outflow) did not show Fos-activity. Stress- and pain-sensitive cortical/subcortical areas, neurons in the limbic system, the hypothalamus and the circumventricular organs were also affected by 4/6NX. Administration of losartan and more strongly moxonidine modulated most effects and particularly inhibited Fos-activity in locus coeruleus neurons. In conclusion, 4/6NX elicits high activity in central sympathetic, stress- and pain-related brain areas as well as in the limbic system, which can be ameliorated by losartan and particularly by moxonidine. These changes indicate a high sensitivity of CNS in initial stages of CKD which could be causative in clinical disturbances.

## Introduction

Patients with the most severe stage of chronic kidney disease (stage 5 CKD) or on maintenance hemodialysis are afflicted by remarkable cognitive deficits [1], [2], [3], [4], enhanced perception of pain [5], [6], sleep disturbances including sleep apnea [6], [7] depression [8], [9], and impaired quality of life [10]. Among the pathogenic factors involved in the disturbances of the central nervous system (CNS) enhanced blood levels of uremic neurotoxins (guanidino- and phenolic-compounds, indoxyl sulphate) [11], neurotoxic advanced glycation end products (AGEs) [12], [13], [14], homocysteine [15], asymmetric dimethylarginine (ADMA) [15], [16], pro-inflammatory cytokines and reactive oxygen species (ROS), which are partly released from the damaged kidney (“distant renal effects“) play a role [17], [18], [19]. Moreover, over-activity of sympathetic nervous system (SNS) [20], [21] contribute to dysregulation of brain functions. In CKD rats, the concentrations of norepinephrine in posterior hypothalamic nuclei and locus coeruleus (LC) are enhanced [22]. The damaged kidneys releases norepinephrine and angiotensin II [23]. Norepinephrine production is further enhanced by accumulated ADMA [24] and reactive oxygen species [25]. Rats with severe acute kidney injury display markedly elevated neuronal *c-fos* and *Fra-2* immunoreactivities in the central biogenic amine cell groups, stress-sensitive forebrain areas/nuclei, central autonomic and neuronal cell groups involved in the regulation of fluid and electrolyte homeostasis [26].

Interestingly, symptoms of cognitive impairment, poor learning, concentration deficits [27], [28], [29], enhanced perception of pain and sleep disorders/sleep apnea [30], [31], [32] may occur even in mild to moderate stages of CKD (GFR <60 ml/min). Recent investigations revealed manifestation of cognitive dysfunction in albuminuric subjects even with a normal GFR [33]. Since albuminuria is a marker of micro-vascular disease it is assumed that it indicates endothelial dysfunction also in brain microvessels. Albuminuria may additionally be associated with accumulation of ADMA [34] and oxidative stress [35].

The underlying pathomechanisms of CNS disturbances in mild to moderate CKD are poorly understood and the potential changes of neuronal activity in the CNS have not been analyzed. Therefore, we investigated the brain activation pattern by Fos after renal mass reduction employing 4/6-nephrectomy (4/6NX). This model assures symmetry in renal afferents signaling to the brain. Due to compensatory renal hypertrophy it is not associated with a relevant rise of uremic solutes or severe hypertension at least in early stages [36]. Therefore the potential direct/indirect effects of damaged kidney on the brain can be studied. Immunohistochemical detection of Fos-expression is a common marker of neuronal activation in CNS [37], [38]. The c*-fos* gene has short expression time, but repeated or permanent chronic stimuli may induce Fos expression persisting for days to weeks [39], [40], [41]. Long-term Fos-immunoreactivity could reflect the re-expression of Fos in neurons that receive continuous input [42], [43].

To study the therapeutic effects on central neuronal activation, the 4/6NX rats were administered the angiotensin II type 1 receptor blocker (ARB) losartan [44], [45], [46], and the central sympatholytic agent moxonidine, a mixed α2-adrenergic and imidazoline receptor agonist [47] in the rostral ventrolateral medulla of the brainstem [48]. Reduced central sympathetic outflow and thereby lowered peripheral sympathetic tone result in protective reno- and cardiovascular effects [48], [49].

## Methods

### Animal Treatment

The investigation was conducted according to the guidelines for studies using laboratory animals (86/609/EEC), after approval of the protocol by the State Veterinary and Food Administration (Bratislava, Slovakia). Male Wistar rats (Charles River, Budapest, Hungary) weighing 284 g ±20 g, were housed in rooms with constant temperature and humidity, 12 h/12 h light/dark cycle, with *ad libitum* access to drinking water and food (SP1, Horné Dubové, Slovakia).

One week after acclimatization, 12 rats were subjected to ablation of 2/3 of the renal parenchyma of the left kidney, followed by a 2/3 nephrectomy of the contralateral kidney 14 days thereafter (4/6NX) [50], both under i.p. ketamin/xylazin anesthesia. Sham (SHAM, n = 4) rats were operated in parallel. Five days after the second surgery, the 4/6NX rats were randomized into 3 groups (n = 4, each) receiving placebo (vehicle), an angiotensin receptor blocker losartan (3 mg/kg body wt/d), or a selective imidazoline receptor-1 agonist, moxonidine (2 mg/kg body wt/d) in drinking water for 60 days. Previous studies showed that these dosages exert renoprotective effects without a relevant antihypertensive action [51], [52]. Since moxonidine is poorly soluble in water, solutions of both drugs were prepared by dilution of the appropriate amount of the substance (adjusted weekly to body weight) in 10 ml of 0.9% NaCl, pH = 4.6, which was added to 990 ml of tap water. The vehicle contained the corresponding amount of pH-adjusted NaCl. One week prior to sacrifice, heart rate and blood pressure were recorded using tail plethysmography. Five days before sacrifice, rats were placed into metabolic cages for 24-hours stool-free urine collection. At sacrifice, blood was collected from the abdominal aorta. Then the rats were cannulated through the left ventricle and right carotid artery, while the brain was slowly perfused with chilled saline to remove the blood from the vessels, and fixed with the chilled infusion of 4% paraformaldehyde. The kidneys were harvested and weighed.

Standard blood and urine chemistry parameters were measured (Vitros 250 analyzer, J&J, Rochester, USA). Plasma concentration of AGE-associated fluorescence [53], and of advanced oxidation protein products (AOPPs) [54] were determined.

### Statistical Analysis of Morphometric and Biochemical Data

All values are expressed as mean ± standard error. Statistical significance among multiple groups was tested using the Kruskal-Wallis test with post-hoc Mann-Whitney U-test (two-tailed, exact) with Bonferroni correction. p<0.05 was considered significant. SPSS v. 16 programs was employed.

### Fos Immunostaining and Semi-quantitative Analysis of the Data

Fos immunostaining was performed on coronal serial sections of rat brains, as previously described [26]. More than 100 different brain areas and nuclei have been checked under the microscope in each animal (n = 16). In more than 50% of the investigated brain areas, neuronal cells did not show any Fos-immunopositivity at the moment of the decapitation. In total, 37 brain areas or nuclei were found that contained Fos-positive neurons in various densities.

The percentage of Fos-immunopositive cells in the total cell number of the investigated brain nuclei or areas were counted and classified. According to the percentual rate of Fos-positive neurons within a brain nuclei, grading 0 to 3 was used, as follows: 1) high Fos expression (grade: 3.00) = more than the half of the cells in the investigated brain areas or nuclei showed Fos-positivity; 2) moderate Fos-expression (grade: 2.00) = the percentage of the Fos-positive cells was between 25 and 50% of the total cell number; 3) low Fos expression (grade: 1.00) = less than the 25 percent of the cells were immunostained for Fos; 4) no Fos expression = grade 0. In the tables, the average values from 3 or 4 animals are given. According to the functional role of the cell groups and brain areas, the Fos-immunoreactive cells were classified into 6 groups: 1) biogenic amine-expressing cell groups, 2) brain nuclei involved in the central regulation of the salt and electrolyte homeostasis, 3) stress-related hypothalamic areas and nuclei, 4) pain-related brain areas and nuclei, 5) limbic brain areas and nuclei, 6) other brain areas and nuclei.

## Results

At sacrifice, the untreated and treated groups did not differ significantly in body weight or kidney-to-body-weight ratio (Table 1). The placebo-administered 4/6NX rats displayed a higher systolic blood pressure (149±14) in comparison to the sham-operated control rats (128±9) (+16%, n.s., Table 1). The high standard deviation in the 4/6 NX rats is a consequence of one outlier with blood pressure of 174 mm Hg. If this rat is excluded, the mean blood pressure of this group would drop to 134±7 Hg. The placebo-administered 4/6NX rats also displayed a lower creatinine clearance (−26%, n.s., Table 1) compared to sham-operated animals. Treatment with losartan or moxonidine did not affect these parameters significantly (Table 1).

**Table 1:** Characteristics of sham-operated and 4/6-nephrectomized (4/6NX) rats at sacrifice.

	Sham-operated	4/6NX			
	Placebo (n = 4)	Placebo (n = 4)	Losartan (n = 4)	Moxonidine (n = 4)	p (K-W)
Body weight (g)	494±45	495±13	438±41	446±21	NS
Kidney/body weight (mg/g)	6.0±0.1	6.4±0.5	5.8±0.3	5.8±0.3	NS
Blood pressure (mm Hg)	128±9	149±14	131±5	129±2	NS
Cl_Creatinine_ (ml/min/1g kidney)	0.86±0.13	0.68±0.03	0.86±0.09	0.74±0.09	NS
Proteinuria (mg/µmol crea)	175±30	603±205^+^	174±14*	307±54	0.024
Serum AGE/Albumin (AU/g)	4.4±0.3	6.3±0.7^+^	6.1±0.1	5.1±0.1*	0.048
Serum AOPPs/Albumin (µmol/g)	4.5±0.5	5.8±1.3	2.4±0.7*	5.5±0.2	0.039

Cl: clearance; AGE: advanced glycation end products-associated fluorescence of plasma; AU: arbitrary units; AOPPs: plasma advanced oxidation protein products; K-W: Kruskal-Wallis test;

+: p<0.05 vs. SHAM-PLAC;

* : p<0.05 vs. 4/6NX-PLAC (Exact 2-sided Man-Whitney U-test with Bonferonni correction).

4/6NX placebo-treated rats revealed a 3.4-fold higher proteinuria (p<0.05) compared to their sham-operated counterparts (Table 1). This was reduced by losartan (−70%, p<0.05), while the moxonidine-induced amelioration (−50%) did not reach significance. Mean food intake was similar among the groups (data not shown), thus proteinuria was not affected by different protein-caloric intake.

Plasma AGE-associated fluorescence was higher (+43%, p<0.05), while that of advanced oxidation protein products (AOPPs) showed a trend to elevated levels (+29%, n.s.) in the 4/6NX rats (Table 1). Administration of moxonidine, but not losartan lowered the AGE levels (−20%, p<0.05), while losartan, but not moxonidine, ameliorated the accumulation of AOPPs (−60%, p<0.05). Plasma levels of albumin were within the normal range in all groups and were not affected by the treatments (data not shown).

### Fos-activation in the Brain of Sham-operated and 4/6NX Rats

In general 4/6NX rats established increased Fos-activation throughout the brain except for few brain nuclei, where no alteration or even decreased activations were observed.

#### Actions on central monoaminergic neurons

**Figure 1:**
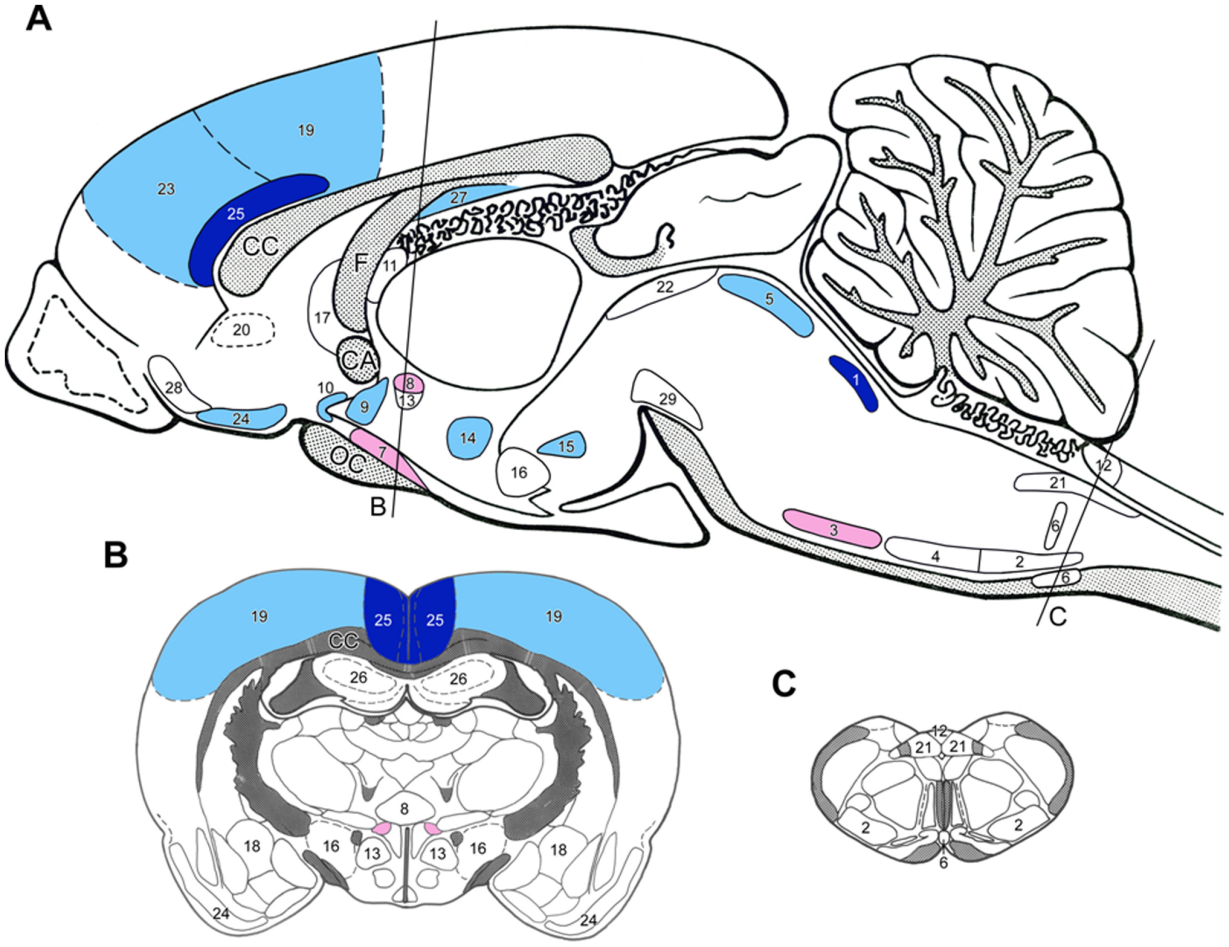
Sagittal (A) and coronal (B and C) drawings of the rat brain with the topography of brain nuclei and areas where neurons responded to experimental chronic renal failure (*4/6 nephrectomy)* with moderate to high expressions of Fos as compared to sham-operated animals. The rostro-caudal levels of the coronal sections are indicated on Fig. A). *Dark blue* = highly, *light blue = *moderately *elevated* Fos activation, *red = *highly, *light red* = moderately *depleted* Fos activation. (For the level of numerical changes *see* Table 2.) *Abbreviations:* CA – anterior commissure, CC – corpus callosum, F – fornix. *Brain nuclei and areas:* 1 – locus coeruleus, 2 – A1 noradrenaline cell group, 3 – A5 noradrenaline cell group, 4 – C1 adrenaline cell group, 5 – dorsal raphe nucleus, 6 – medullary raphe nuclei, 7 – supraoptic nucleus, 8 – hypothalamic paraventricular nucleus (magnocellular portion), 9 – preoptic AV3V area, 10 – organum vasculosum laminae terminalis (OVLT), 11 – subfornical organ, 12 – area postrema, 13 – hypothalamic paraventricular nucleus (parvocellular portion), 14 – hypothalamic dorsomedial nucleus, 15 – supramamillary nucleus, 16 – lateral hypothalamic area, 17 – lateral septum, 18 – central amygdala nucleus, 19 – somatosensory cortex, 20 – viscerosensory cortex (insula), 21 – nucleus of the solitary tract, 22 – periaqueductal gray matter (PAG), 23 – prefrontal cortex, 24 – piriform cortex, 25 – anterior cingulate cortex, 26 – hippocampus (CA1-3), 27 – subiculum, 28 – olfactory tubercle, 29 – substantia nigra/VTA dopamine neurons.

Slight to moderate Fos-activity was detected in some of the monoaminergic neurons in the lower brainstem of sham-operated rats. 4/6NX resulted in further activation in the major cell groups such as locus coeruleus (LC), dorsal raphe and tuberomamillary nuclei, while neurons in several other (smaller) groups remained silent (Fig. 1, Table 2).

**Table 2:** Fos expression in various brain areas after 4/6NX and its treatment with losartan and moxonidine.

	Sham-operated rats (Grade)	4/6NX rats					
		Placebo		Losartan		Moxonidine	
		Grade	Δ vs. Sh-P	Δ vs. 4/6NX-P	Δ vs. Sh-P	Δ vs. 4/6NX-P	Δ vs. Sh-P
**Biogenic amine cell groups**							
locus coeruleus	0.75	2.00	**+1.25**	−*1.25*	0	−*1.75*	−*0.50*
A1 NA cell group	0	0	0	0	0	0	0
A5 NA cell group	1.00	0.25	−*0.75*	0	−*0.75*	0	−*0.75*
C1 A cell group	1.00	1.00	0	0	0	0	0
substantia nigra/VTA	0	0.25	**+0.25**	0	**+0.25**	0	**+0.25**
dorsal raphe nucleus	0.25	0.75	**+0.50**	−*0.50*	0	0	**+0.50**
medullary raphe nuclei	1.00	1.00	0	0	0	−*0.50*	−*0.50*
tuberomamillary nucleus	2.00	2.50	**+0.50**	−*0.75*	−*0.25*	−*0.50*	*0*
**Salt and water homeostasis**							
supraoptic nucleus	1.50	1.00	−*0.50*	0	−*0.50*	0	−*0.50*
magnocellular PVN	1.00	0.25	−*0.75*	0	−*0.75*	**+1.50**	−*0.75*
preoptic AV3V area	0.50	1.00	**+0.50**	0	**+0.50**	0	**+0.50**
**Blood-brain barrier free**							
OVLT	1.00	1.50	**+0.50**	0	**+0.50**	−*0.50*	0
subfornical organ	0	0	0	**+0.75**	**+0.75**	0	0
area postrema	0	0	0	0	0	0	0
**Stress-related brain areas**							
parvocellular PVN	1.75	1.75	0	0	0	−*0.50*	−*0.50*
dorsomedial nucleus	1.25	2.00	**+0.75**	0	**+0.75**	−*0.50*	**+0.25**
supramamillary nucleus	1.50	2.25	**+0.75**	−*0.50*	**+0.25**	−*1.00*	−*0.25*
lateral hypothalamic area	1.25	1.50	**+0.25**	0	**+0.25**	−*1.00*	−*0.75*
lateral septal nucleus	2.00	2.25	**+0.25**	−*1.25*	−*1.00*	−*0.50*	−*0.25*
central amygdala	0	0	0	0	0	0	0
**Pain-related brain areas**							
somatosensory cortex	1.50	2.50	**+1.00**	−*0.75*	**+0.25**	−*1.50*	−*0.50*
viscerosensory cortex	1.25	1.50	**+0.25**	**+0.25**	**+0.50**	−*0.50*	−*0.25*
nucl. of the solitary tract	0	0	0	0	0	0	0
PAG (central gray), dorsolat.	0.50	0.50	0	0	0	0	0
**Limbic brain areas**							
prefrontal cortex	1.00	2.00	**+1.00**	**+0.50**	**+1.50**	−*1.25*	−*0.25*
piriform cortex	2.25	2.50	**+0.25**	0	**+0.25**	−*1.25*	−*1.00*
anterior cingulate cortex	0.50	1.50	**+1.00**	−*0.50*	**+0.50**	−*1.25*	−*0.25*
Hippocampus (CAs+DG)	0.50	0.75	**+0.25**	0	**+0.25**	0	**+0.25**
subiculum	0.25	1.00	**+0.75**	−*0.75*	0	−*0.75*	0
olfactory tubercle	1.00	2.00	**+1.00**	0	**+1.00**	−*1.00*	0

P: placebo; Grade: grading of c-fos expression (as given in Methods section); Δ: change; Sh: sham-operated; NA: noradrenaline; A: adrenaline; VTA: ventral tegmental area; PVN: paraventricular nucleus; OVLT: organum vasculosum laminae terminalis; PAG: periaqueductal gray matter; dorsolat.: dorsolateral; CAs: anterior commissure; DG: dentate gyrus.

*Noradrenaline cell group,* fairly high Fos-activity was seen in LC neurons of 4/6NX rats, while the forebrain projecting caudal ventrolateral (A1 cell group) and dorsomedial (A2 cell group) noradrenaline neurons did not show altered activities. Neurons projecting to the sympathetic preganglionic neurons in the spinal cord (A5 noradrenaline cell group) showed even less activity in 4/6NX rats than in sham-operated animals.

*Adrenaline* neurons in the medulla oblongata (C1-C3 cell groups), or *dopaminergic* neurons in substantia nigra/ventral tegmental area and in diencephalon showed minor or no activation in 4/6NX rats (Table 2).

#### Actions on brain nuclei involved in the central regulation of the fluid and electrolyte homeostasis and on brain areas where no blood-brain barrier exists

In sham-operated rats the Fos-activation was moderate to high in the vasopressin-expressing magnocellular neurons of the supraoptic and paraventricular hypothalamic nuclei (Table 2) which was reduced by 4/6NX (Fig. 1). Elevation in Fos-activity was observed in the anterior (preoptic) part of the hypothalamus, namely in the median preoptic antrioventral third ventricle region (AV3V region) and in the organum vasculosum laminae terminalis (OVLT) of the 4/6NX rats (Table 2). 4/6NX did not elicit Fos-activation in the other blood-brain barrier-free brain areas (subfornical organ-SFO-, area postrema).

#### Actions on “stress-related” hypothalamic areas and nuclei

Neurons in all “stress-sensitive” hypothalamic nuclei (parvocellular paraventricular, supramamillary, dorsomedial) and in the lateral hypothalamic area showed signs of activation in sham-operated rats with further Fos-activation in 4/6NX animals (Fig. 1, Table 2). Elevated Fos-activity was also found in the lateral septal nucleus in 4/6NX rats (Fig. 1), nevertheless, neurons in the central amygdaloid nucleus failed to show any sign of Fos-expression either in sham-operated or 4/6NX rats (Table 2).

#### Actions on pain-related brain areas

Strong Fos-activation was detected both in somato- and viscera-sensory (insular) cortical areas in sham-operated, and stronger in 4/6NX animals. In contrast, subcortical neurons in pain-related medullary viscerosensory nucleus (the nucleus of the solitary tract - NTS) or in the periaqueductal central gray (PAG) did not express Fos in 4/6NX rats (Fig. 1, Table 2).

#### Actions on limbic cortical and subcortical areas

Neurons in the major limbic cortical areas (prefrontal, piriform, anterior cingulate cortices) were strongly activated in both sham-operated and 4/6NX animals (Fig. 1, Table 2). In the hippocampus, no Fos-activation was seen either in the CA1-CA3 regions or in the dentate gyrus, while slight activity was observed in the ventral subiculum of 4/6NX rats. Strong Fos-activation was seen in the olfactory tubercle (Fig. 1).

### Fos Activation in the Brain after Losartan and Moxonidine Treatments in 4/6NX Rats

In general, losartan or moxonidine treatments altered Fos-expression throughout the CNS and showed many similarities.

#### Actions on central monoaminergic neurons

**Figure 2:**
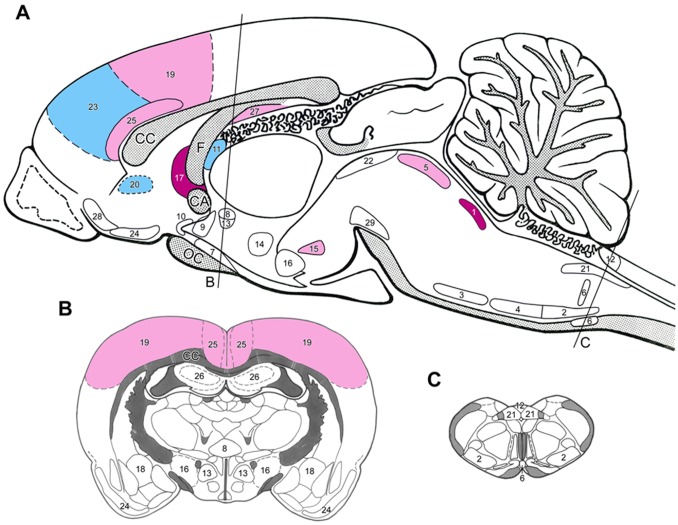
Sagittal (A) and coronal (B and C) drawings of the rat brain with the topography of brain nuclei and areas where *nephrectomized rats* responded with *losartan treatment* with moderate to high expressions of Fos as compared to *placebo-treated nephrectomized* animals. The rostro-caudal levels of the coronal sections are indicated on Fig. A). *Dark blue* = highly, *light blue = *moderately *elevated* Fos activation, *red = *highly, *light red* = moderately *depleted* Fos activation. (For the level of numerical changes *see* Table 2.) *Abbreviations and numbers: see* Fig. 1.

**Figure 3:**
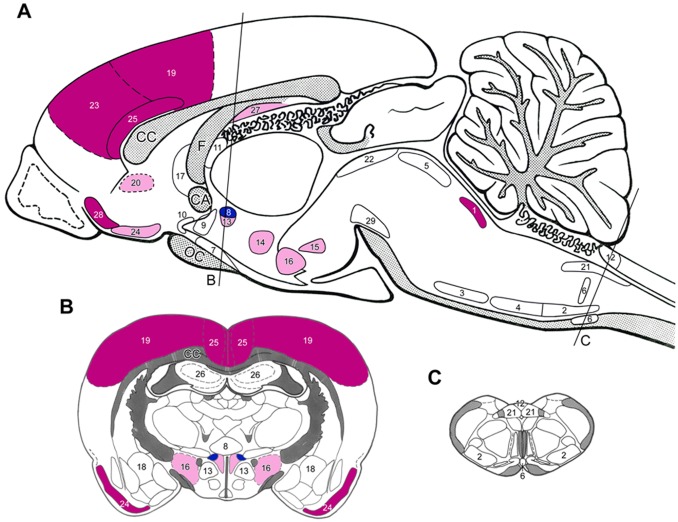
Sagittal (A) and coronal (B and C) drawings of the rat brain with the topography of brain nuclei and areas where neurons in *nephrectomized rats* responded to *moxodinine treatment* with moderate to high expressions of Fos as compared to *placebo-treated nephrectomized* animals. The rostro-caudal levels of the coronal sections are indicated on Fig. A). *Dark blue* = highly, *light blue = *moderately *elevated* Fos activation, *red = *highly, *light red* = moderately *depleted* Fos activation. (For the level of numerical changes *see* Table 2.) *Abbreviations and numbers: see* Fig. 1.

*Losartan* and *moxonidine* markedly reduced the enhanced Fos-activation in the LC of the 4/6NX rats (Fig. 2 and 3, Table 2). In addition, moxonidine lowered the moderate Fos-activation in noradrenaline (A5) and adrenaline (C1) neurons in the ventrolateral medulla that project to the spinal sympathetic preganglionic neurons (Fig. 3). It also reduced Fos-activity in medullary raphe neurons that project to the spinal cord of 4/6NX rats, whereas the forebrain projecting serotonin neurons in the dorsal raphe nucleus were not activated (Table 2). The fairly high numbers of Fos-positive cells in the histaminergic tuberomamillary nucleus were reduced by losartan (Table 2).

#### Actions on brain nuclei involved in the central regulation of the fluid and electrolyte homeostasis and on brain areas where no blood-brain barrier exists

The moderate Fos-activity in supraoptic and magnocellular paraventricular nuclei was slightly elevated (Table 2). Moxonidine had minor effect on supraoptic nuclei, but markedly increased Fos-activity in paraventricular nucleus (Table 2, Fig. 3). Losartan did not modulate Fos-activity in OLVT (Fig. 2, Table 2), but monoxidine reduced the activity of these neurons (Table 2). It is hard to explain the excitatory action of losartan on the SFO. Both drugs failed to elicit any such effect in area postrema (Table 2).

#### Actions on “stress-related” hypothalamic areas and nuclei

In 4/6NX animals the number of Fos-positive neurons in the stress-related nuclei of the hypothalamus was much lower after losartan or moxonidine treatment than that of placebo-treated rats (Table 2). Both drugs failed to act on central amygdaloid neurons.

#### Actions on pain-related brain areas

In 4/6NX rats, both drugs strongly reduced the number of Fos-positive neurons in the somatosensory cortex. They had opposite effects on the viscerosensory cortex (insula): losartan further activated Fos, while moxonidine reduced it (Table 2, Fig. 2 and 3).

#### Actions on limbic cortical and subcortical areas

The 4/6NX-induced high number of Fos-positive cells in the investigated limbic cortical areas and the olfactory tubercle were diminished after moxonidine treatment (Fig. 3, Table 2). The response to losartan treatment was ambivalent. Some areas like the prefrontal cortex, showed further increase in the number of Fos-positive cells, while their number decreased in the anterior cingulate cortex and in the subiculum (Table 2, Fig. 2). Both drugs did not modulate Fos-activity in the hippocampus (Table 2).

## Discussion

Here, we demonstrate for the first time that 4/6NX elicits marked alterations of Fos-activation in different brain areas/nuclei. Due to compensatory renal hypertrophy the GFR was only mildly reduced, but proteinuria and circulating AGEs were elevated. The higher blood pressure in the 4/6 NX rats was in particular due to one outlier animal with hypertension, while the blood pressure in other animals of the group was not elevated. Therefore, it seems unlikely that the observed alterations in neuronal activation are particularly induced by hypertension. However, an ultimate statement about the potential role of hypertension on central neuronal activity requires an additional study employing hypertensive/normotensive animals with or without 4/6NX.

Renal function of the 4/6NX rats corresponded clinically to CKD stage 2–3a. This stage in humans may be associated with an enhanced prevalence of pain perception and sleep disturbances [6], [31]. In addition, in humans in the presence of higher levels of albuminuria/proteinuria a cognitive decline may occur even with preserved GFR [33], [55].

As a sign of central sympathetic overactivity, the 4/6NX rats displayed a moderate to high Fos-activity in the neurons of LC, which project to the forebrain and innervate the cerebral cortex, the limbic system, the thalamus and the hypothalamus. LC is involved in many functions such as arousal, attention and stress. However, its prolonged activation could result in a disorganized and unfocused behavior [56]. No alteration in Fos-activity was observed in the A5 noradrenaline and C1 adrenaline cell groups, as well as in parvocellular neurons in hypothalamic paraventricular nucleus, that innervate sympathetic preganglionic neurons in spinal cord and affect peripheral sympathetic outflow. In patients with CKD peripheral sympathetic overactivity is a prevalent disturbance. Its severity is inversely related to eGFR and can be observed even in moderate CKD [21], [24], [57], [58], [59], [60].

The dorsal raphe serotonin-expressing neurons, which provide a significant proportion of the serotonin innervations to the forebrain, showed a moderate activation in the 4/6NX rats. In humans, serotonin pathways are namely involved in mood, memory processing, sleep, cognition and satiety. The observed Fos-activation in the prefrontal and anterior cingulate cortex may be implicated in depressive symptoms and sleep disorders occurring in mild to moderate CKD [31], [32], [61]. In advanced CKD in rats and humans the high brain serotonin levels could contribute to uremic anorexia [62], [63], [64].

Our data suggest that histamine-expressing neurons in the hypothalamic tuberomamillary nucleus (the major if not the sole site of histamine synthesis in the brain) become activated in mild CKD. This system has a key position in basic body functions such as sleep/waking cycle, learning and synaptic plasticity [65]. In advanced CKD in rats and patients the high brain histamine content may contribute to an increased vascular permeability, brain edema and disruption of blood-brain barrier [66], [67].

Our data suggest that renal mass ablation elicits also changes in the regulation of fluid and salt homeostasis. Fos-activity of the vasopressin-expressing supraoptic and magnocellular paraventricular neurons is reduced, while neurons in the preoptic AV3V area and the OVLT, where the majority of brain atrial natriuretic peptide (ANP)-expressing cells are located [68], showed moderate Fos-activity. Accordingly, in earlier studies rats with advanced CKD presented elevated levels of ANP in the OVLT and the AV3V area [69].

In stress-related brain areas/nuclei a high Fos-activation was observed in the dorsomedial and supramamillary hypothalamic nuclei, but unexpectedly no changes occurred in parvocellular PVN and in central amygdala. In CKD patients with an eGFR ≤60 ml/min an exaggerated hemodynamic response to mental stress has been observed [70].

Among the pain-related brain areas Fos-expression was upregulated in the somatosensory and viscerosensory cortex, indicating central sensitization to noxious factors including enhanced stress-induced hyperalgesia in animal models [71]. 4/6NX failed to elicit Fos-activation in NTS and PAG.

In the limbic brain areas Fos-activity was augmented in all six investigated areas/nuclei. The prefrontal cortex is considered to be implicated in cognitive control and “the ability to orchestrate thoughts and actions in accordance to internal goals” [72]. In humans neurons of anterior cingulate cortex are involved in regulation of blood pressure, heart rate and baroreflex sensitivity [73] as well as in empathy and emotion [74].

A marked expression of *c-fos* was found in the olfactory bulb of 4/6NX rats. In humans disturbances in taste and smell function are uncommon in mild CKD but may appear in moderate to severe CKD [75], [76], [77] and may contribute to appetite loss [77], [78], [79].

The observed changes in Fos-activity in various brain areas do not allow an extrapolation of these findings on behavior, sleep disturbances or pain perception. There is a need to perform an appropriate battery of available behavioral tests in rats subjected to 4/6 NX as well as sham-operated rats, which could bring an insight on the association of the observed changes in brain signaling with those of behavior.

In the 4/6NX rats administration of moxonidine and losartan exerted marked effects on Fos-activity. A subantihypertensive dose of moxonidine caused a substantial reduction of Fos-activity in the LC and all investigated forebrain areas/nuclei innervated by LC neurons. This corresponds to earlier studies in 5/6NX rats, where moxonidine reduced the central sympathetic overactivity [20]. The effects of moxonidine were particularly striking in all stress-related areas/nuclei. Lowering of Fos-activity was very pronounced in the dorsomedial nucleus, the supramamillary nucleus and in the hypothalamic area. As expected, moxonidine also decreased Fos-activity in pain-related areas such as somato- and viscerosensory cortex. These findings fit to clinical observations in humans that agonists of α-2-adreno-receptors (which mediate analgesia) are very effective in the treatment of pain [80]. Moxonidine also reduced the enhanced Fos-activity in 5 of the 6 investigated limbic brain areas: prefrontal, piriform cortex, anterior cingulate cortex, the subiculum and olfactory tubercle.

In the areas/nuclei involved in fluid and salt homeostasis moxonidine decreased Fos-activity in supraoptic and magnocellular PVN, where the hormones oxytocin and vasopressin are formed. Fos-activity in the preoptic AV3V region was increased as compared to sham-operated rats. Acute moxonidine administration enhances the ANP formation resulting in diuresis and natriuresis in rats [81].

Losartan, which passes through the blood-brain barrier [60] and ameliorates central sympathetic activity [21], [55], [82], [83] reduced Fos-activity in particular in LC and in the stress-related brain areas such as supramamillary and lateral septal nuclei. In pain-related areas losartan lowered Fos-activity only in the somatosensory cortex, while the activity in the viscerosensory cortex even increased. The latter effect fit to the clinical observation of a hyperalgesic action of losartan as well as of the ACE-inhibitor enalapril in the treatment of hypertensive patients [84]. Also in the limbic brain areas the response to losartan was different to that of moxonidine: elevation of Fos in prefrontal cortex and lowering in anterior cingulated cortex and subiculum. Among the nuclei involved in fluid and salt homeostasis, losartan enhanced the Fos-activity in SFO which is involved in blood volume homeostasis.

Which mechanisms underlie the altered Fos-activation pattern in mild CKD? Recent cross-sectional and longitudinal studies in humans [85] documented that albuminuria may be associated with cognitive decline [86]. In the PREVEND study particularly young subjects with albuminuria presented a poorer cognitive function [33]. In other studies, it has been shown that albuminuria is associated with endothelial dysfunction [87] and cerebral small vessel disease [88]. Therefore it is conceivable that the cognitive impairment may be a consequence of sub-clinical cerebral micro-vessel disease. Extravasation of proteins due to microvascular disease could contribute to brain damage as demonstrated by white matter hyperintensities with a perivascular distribution [89], [90]. In line with these findings administration of centrally acting ACE-inhibitors in older adults concurrently ameliorated not only proteinuria but also protected against cognitive decline [91]. Correspondingly in our study administration of losartan and moxonidine was associated with a decline in proteinuria.

A possible link between albuminuria, endothelial dysfunction and cognitive decline in humans could be an enhanced formation of ADMA [92]. Remarkably, serum ADMA levels are directly related to albuminuria and may be its causal contributor [24]. ADMA attenuates NO-dependent vasodilation, enhances oxidative stress, triggers sympathetic over-activity and promote atherosclerosis in rats [93]. In healthy subjects administration of subpressor doses of ADMA decreased cerebral perfusion and increased vascular stiffness [94]. In the Framingham offspring study higher plasma ADMA levels were associated with MRI markers of vascular brain injury [95]. Therefore, ADMA may be involved in the pathogenesis of cerebrovascular disease and the concomitant cognitive decline in CKD [94]. ADMA also promotes progression of non-diabetic kidney diseases in humans [16]. The potential involvement of ADMA in the above mentioned conditions is supported by the observation that the ACE-inhibitor, ramipril, lowers proteinuria and circulating ADMA in patients with diabetic nephropathy [96]. We hypothesize that in our study the losartan- and moxonidine-induced modulation of neural activation in the 4/6NX rats is in part a consequence of an improved endothelial dysfunction as a consequence of decreased ADMA levels. Moreover, ameliorated Fos-activity are explained by specific effects of losartan and particularly moxonidine on the central sympathetic nervous system. The strength of the current study is the first description of numerous central disturbances in the early stage of CKD. Its limitation is the small size of the animal groups.

In conclusion, 4/6 ablation of renal mass alters the activation of neurons in numerous brain areas/nuclei with different functional properties. Especially, the central sympathetic nervous system such as the LC, the limbic areas as well as stress- and pain-related areas are affected. It is conceivable that these activations may be causative or predictive of numerous clinical complications. Treatment with the ARB losartan or the central sympatholytic agent moxonidine ameliorates many of the observed central alterations.
